# Personal assistants in England and the factors associated with absenteeism

**DOI:** 10.3389/fpubh.2022.970370

**Published:** 2022-10-10

**Authors:** Daniel Roland, Stephen Allan, Eleni Chambers, Debs Smith, Katerina Gousia

**Affiliations:** ^1^Personal Social Services Research Unit, School of Social Policy, Sociology and Social Research, University of Kent, Canterbury, United Kingdom; ^2^Patient and Public Involvement Research Advisor, Division of Nursing & Midwifery, Health Sciences School, University of Sheffield, Sheffield, United Kingdom; ^3^Patient and Public Involvement Research Advisor, Public Involvement Research Advisor Network, Personal Social Services Research Unit, University of Kent, Canterbury, United Kingdom

**Keywords:** long-term care, personal assistants, domestic care workers, absenteeism, older people, home care, sick leave

## Abstract

Personal assistants (PAs) have become an increasingly important element of long-term care (LTC) in England since the introduction of Direct Payments in 1996 and the Care Act 2014 legislation. The PAs, who are directly employed by social care users, can perform a number of support tasks including vital assistance in activities of daily living (ADL). Internationally these roles would be classed as domestic care work, including the employment of migrant care workers, e.g. in Germany and Austria. High turnover rates and work absenteeism in this market can cause disruption of these important daily activities, causing LTC users to potentially suffer neglect and poorer quality of life. Although there is research on turnover and absenteeism in nursing workforce in hospitals and LTC workers in nursing homes, little attention has been given to reasons for turnover of PAs and even less for absenteeism, which often precedes turnover, in a workforce of over 100,000 people in England. This research aims to fill this gap in knowledge by analyzing the reasons behind the absenteeism of PAs using quantitative methods. We used survey data of PAs in England, exploring the factors associated to one form of absenteeism—sick leave from work. After controlling for a number of factors ranging from job characteristics such as number of hours worked and type of contract, socio-economic characteristics from the PA and their employer, and supply and demand factors at local government region, the findings suggest a number of factors that significantly influenced sick leave, including distances traveled to work and number of PAs employed. Following the analysis, two people with life experience of LTC discuss the findings of the study and how they compare to their experiences of the market for PAs, providing a unique perspective from the people who could benefit the most from improving PA retention and reducing absenteeism.

## Introduction

Increasingly many countries deliver long-term care (LTC) at home. This choice, as opposed to the more costly institutionalized care, aims to meet the needs of the aging population of countries (1) while guaranteeing quality of life in accordance with principles outlined by the United Nations (2). Moreover, this choice respects the preferences of those requiring LTC support as they find it more comfortable staying in familiar surroundings (3, 4). Limiting public social care expenditure has been pursued by many countries and the motto “aging in place” at home fits well with this goal (5).

Countries such as UK, Austria, France, Finland, Germany and USA, among others, provide benefits in cash to help older people pay for home care services (6–8). Even China, a country that until recently enjoyed a relatively young demographic structure and culturally expects family to care for their elders, has implemented pilot programs for long-term care insurance (LTCI) that include cash benefits in some cities as a potential policy instrument to deal with the demographic change that will see the proportion of people over 65 years old increase from 13% in 2020 to 27.9% by 2050 (9, 10). In the UK, current legislation has enabled LTC users to directly employ Personal Assistants (PAs) by using direct payments to best support their LTC needs. Initially set in the ‘Direct Payments Act 1996', the take-up of direct payments started slowly but increased over the years going from 65,000 in 2008 to 230,000 people receiving the benefit in 2020 (11–13). Further, the ‘Care Act 2014' implemented the ‘personal health budget' (PHB), organized by the UK's national health system (NHS), as an additional mean through which people with ongoing health issues can hire staff to meet their health needs. However, there are limitations to what type of work can be done by the PA according to the source of funding, e.g. PHB funds are for health related services only and not social care. The common overlap between health care and social care aspects in LTC means it is sometimes difficult to disentangle the two and many PAs are willing to provide health related care, subject to proper training (14). The focus of this study is in the social care element of LTC, specifically of PAs providing home care, but without ignoring the relevance and importance of health care and the existing body of evidence from care homes and nursing homes.

To be defined as a PA in England, one has to be employed directly by a person who needs support or by a family member or representative of a person who needs support, working directly in a person-centered way to enable them to live their life according to their wishes and interests (13). In other countries, PAs are called personal care workers, caregivers or domestic care workers and other similar names. The definition of their roles also differs from country to country. Our interest is in the domestic care workers as defined in England, where their work can involve supporting care users to perform standard activities of daily living (ADL), such as showering and dressing, but can also include organizing and supporting individuals with their social and physical activities or supporting with tasks around the house such as shopping, cleaning and cooking (15). There are estimates of about 100,000 people employed in 135,000 jobs as PAs providing care to those who receive direct payments (13). It is unknown as to how many people self-fund employment of PAs and how many people receiving PHBs may also employ PAs. Nonetheless, the lower-bound estimate of 100,000 PAs in England represents around 6.5% of the adult social care workforce of 1.54 million (16).

Those directly employing PAs deal with the recruitment and retention of staff. Added to this, any turnover and vacancies for PA staff will necessarily affect the wellbeing of those with LTC needs. In England, turnover of PAs is usually lower than for the care workforce in general. For 2022, PA turnover was estimated to be 18.3%, lower than for care workers in the independent sector which stood at 35.3% (13). This might be explained by the fact that being directly employed by the service users allowed for closer relationships to be formed, thus reducing turnover and absenteeism (17, 18). It might also be due to differences in the work carried out by workers and better terms and work conditions for PAs (13). Even so, the turnover rate for PAs in the UK is higher than for nurses (19).

Although substantial qualitative work has been done exploring the dynamics of PAs and their work and relationships with IEs (20–24), there is currently little quantitative evidence about the factors that affect the recruitment and retention of PAs. Gousia and Allan (25) found that economic factors, including local unemployment rates and alternative social care employment, influenced the PA staff turnover and vacancies faced by service users. This supported previous qualitative evidence for PAs for England (24, 26, 27). There is also limited international research on the economic factors affecting the satisfaction and commitment of domestic care workers. Evidence on Israeli migrant live-in care workers found that the level of needs of the caree, e.g. living with dementia, the level of job control and the relationship between carer and caree affected job satisfaction, burden and intention to leave (28–30). More widely, evidence from the US has found that home care workers' pay and conditions significantly influenced their turnover and intention to leave (31, 32).

In this study, we assessed the economic factors driving absenteeism of PAs. In the economic literature, there is a strong link between absenteeism and other decisions regarding employment, including (voluntary) staff turnover. We describe this link more fully for LTC in the next section, before we present the data, methods and findings of the study. Two people with experience of direct payments then discuss how these findings fit with their view of the PA market and what the findings mean for policy and practice.

## Background

It has long been understood, in all industries, that overall job satisfaction is consistently and inversely associated with job turnover (33, 34) and attempts to understand the causes of both turnover and absenteeism have been widely discussed and theorized since then (35). Similar to other industries, LTC staff, despite altruistic motivations and a vocational view of their jobs (36, 37), are significantly negatively affected in their job satisfaction, likelihood of quitting and wellbeing by low pay (usually at minimum wage), lack of career progression and challenging work conditions. This has direct implications on retention, productivity, work ethics as well as care outcomes and service users' quality of life (38, 39). Adequate pay is seen as an important means for retention of LTC workers (40). As such, there are efforts to establish pay scales in accordance with the level of work required from PAs, their roles and responsibilities, so that they are competitive in comparison with other industries with similar levels of experience and qualifications (41), but this would have to take into account local government LTC budgets. For those that employ PAs to support their health and LTC needs, the direct employment relationship and the pressure that can come with organizing this can add further stresses to the employer/employee roles (12). In some countries, the use of migrant workers presented itself as a solution to shortages in the accessibility and availability of formal care service provision, but this came with other issues such as a hierarchical relationship between care worker and employer, lack of proper training and insecurity about working conditions and legal status (42).

Any form of absence from work that is not planned in advance, including sick leave, is considered absenteeism. The cost of absenteeism for employers in general can be high, and for IEs in particular the loss of a member of staff could have negative implications for their wellbeing and health. In addition, work behaviors, including absenteeism, are likely to be linked to the same precursors that drive employee turnover, such as low commitment and job satisfaction (43). Qualitative research in France in nursing homes indicates that not only did absenteeism lead to a harmful impact on the quality of care received by patients, it was also intertwined with work overload and stress, a deterioration in nurses' attitudes and behaviors, which turned into a harmful spiral (44). Evidence from the Netherlands suggest that absenteeism of nurses in care homes were directly associated with staff health issues only, but signaled that poor or decreasing organizational commitment could lead to reduction in wellbeing and an increase in health complaints, and therefore higher absenteeism (45). In the LTC market in England there is evidence of higher turnover for those employers with higher absence rates, measured by sick days (46). In 2021, on average, PAs in England took 2.2 days of sick leave in the past 12 months, which was almost four times lower than the average number for care workers employed by for-profit and not-for-profit LTC providers (13).

The relevance of understanding turnover and absenteeism in this industry is linked to the size of the markets for PAs internationally, which are large and likely to grow in the future given aging populations. Measuring the exact size of these markets is difficult as many countries do not conduct systematic surveys, with many estimating the overall number of domestic care workers, providing an approximation at best. The definition of PAs and nurses varies across countries, which also contributes to the difficulty in obtaining precise estimates that are comparable across countries. However, it is safe to say that the size of the PA market is considerable in some developed countries as shown in Table 1.

**Table 1:** Domestic care workers market size.

**Country**	**Number of PAs**	**Year**	**Comment**
Austria^a^	12,806	2020	Considering full-time mobile workers only, the actual number of workers is higher.
England^b^	100 thousand	2022	Low estimate considering only carers paid with Direct Payment
Germany^c^	317 thousand	2017	Outpatient nursing carers involved in body care and housekeeping
Netherlands^d^	100 thousand	2019	LTC workers at home, including nurses and personal carers
South Korea^d^	248,269	2019	LTC workers at home, including nurses and personal carers
Switzerland^d^	48,220	2019	LTC workers at home, including nurses and personal carers
USA^d^	1.3 million	2019	LTC workers at home, including nurses and personal carers

*Source*: ^a^(43); ^b^(13); ^c^(44); ^d^(45).

## Data

The data for this study came from the Skills for Care survey of Individual Employers (IEs) and PAs collected in 2019. The Skills for Care (SfC) is the strategic workforce development and planning body for adult social care in England and works with employers, PAs, government and partners to produce reports on the status of the social care workforce and other relevant information essential to understand the key drivers of workforce change through the use of insight, data and evidence (47).

The SfC survey of IEs and PAs was conducted anonymously across England and started in January 2019. Through the use of two national support organizations and an online survey, SfC surveyed nearly 18,500 IEs and their PAs (48). All IEs and their PAs who were in contact with the national support organizations were encouraged to participate. A little over 10% of the surveyed IEs returned a response along with 2,428 PAs, corresponding to roughly 2.4% of the total number of PAs in England. The respondents represented all regions of England and nearly all the local government areas (Local Authorities, 136 out of 152).^1^ The survey focused on IEs in receipt of direct payments but also included IEs funding the payment of their support staff through private self-funding or PHBs provided by the NHS. For the purpose of the survey, PAs were considered to be any social care support worker hired directly by the IE or by their relatives or legal representatives. The data controllers of the secondary data used in this research, Skills for Care, anonymised the data before it was handled by the researchers and the study was part of a wider project which received ethical approval from the University of Kent SRC Ethics Panel (ref SRCEA 240).

There are few published studies with a large number of PAs being surveyed, making comparisons between samples difficult. Nonetheless, other studies with smaller sample sizes such as Woolham et al. (23) and Shakespeare et al. (22) show that the typical PA in England is a white British woman around age 45, very similar to what is shown in the following section. Although the survey may not be representative of domestic care workers in England, the survey data was used as the basis for national estimates of the size of the PA workforce and their pay and hours of work and so can be seen as the most comprehensive data currently available (48). As such, it contained relevant information essential to this study. In the PA survey, personal information was collected such as gender, age, disability, ethnicity, nationality, social care qualifications alongside work information such as basic pay, the number of jobs the PA has, how many years of experience in the role or elsewhere in the social care market, the type of work contract, number of hours worked and the distance necessary to travel to work. The survey also asked PAs for the number of days of sick leave that they had over the last 12 months. The IE survey also collected useful information for this study, specifically their age, the number and types of support needed, the way through which they funded payments for the support staff and the total number of PAs employed. We also linked Local Authority area data with the location information provided in the IE survey, using Job Seeker's Allowance rate, an unemployment benefit, as a proxy for available workforce in the area. As a proxy for the demand for LTC workers in each area, the availability of supply of social care was measured through the number of registered care homes and home care providers according to the registry kept by the Care Quality Commission (CQC).

### Sample and descriptive statistics

Table 2 contains summary statistics of the initial sample of PAs after matching with their respective IEs, a total of 2,304 observations. The majority of PAs are female (83.4%). The average age is 45 years old, most of them are white as only 14.4% declared themselves to be not white. Less than 5% of PAs reported being disabled and slightly less than a fifth of them (18.8%) had more than one job. The average distance that they had to travel to work was 5.88 miles. Most of the care workers had a permanent contract (84.7%) and they had on average a little < 4 years experience in the role (3 years and 9 months), earning on average £9.29 per hour (~EUR 11) working 17.42 hours per week. Most PAs did not take sick leave, as 28.0% reported taking sick leave in the last 12 months taking on average 6.1 days of sick leave for this subsample and an overall average of 1.7 days for the whole sample. About a quarter of IEs had more than one type of support need (26.5%), about three in 10 had a learning disability (29.5%) and a quarter were over 65 years old (24.6%). Most of the IEs were funding the payment of PAs with Direct Payment benefit, with a minority funding it with PHBs or private funds (10.3 and 10.5% respectively). The average IE also employed more than two PAs (2.37) to work for them.

**Table 2:** Summary statistics.

**Variable**	**Mean**	**Standard deviation**	**Observations**
**Personal assistant**			
Sick leave (yes, %)	27.97	44.90	2,052
Days of sick leave	1.70	7.27	2,052
Basic pay rate (British pounds)	9.29	1.70	2,060
Distance from work (miles)	5.88	12.12	1,915
Permanent contract (yes, %)	84.70	36.00	1,915
More than one job (yes, %)	18.80	39.08	2,229
Years in the role	3.81	5.28	2,001
Fixed hours (yes, %)	79.10	40.67	2,124
Hours worked (week)	17.42	17.49	2,076
Female (yes, %)	83.41	37.21	2,212
PA disability (yes, %)	4.23	20.14	2,150
Non-white (yes, %)	14.46	35.18	2,220
Age	45.39	14.27	2,175
**Individual employer**			
More than one type of support need (IE)	26.50	44.15	2,294
IE learning disability (yes, %)	29.56	45.64	2,304
Direct Payment* (yes, %)	81.17	38.69	2,304
Personal Health Budget* (yes, %)	10.33	30.44	2,304
Self-funded* (yes, %)	10.55	30.72	2,304
Total number of PAs employed	2.37	2.22	2,274
IE is 65 years or older (yes, %)	24.58	43.07	2,266
**Local authority**			
JSA allowance (rate of uptake)	0.67	0.45	2,262
Total number of home care providers	86.14	60.93	2,270
Total number of Care Homes	170.34	129.40	2,270

*Source:* Skills for Care survey of Individual Employers 2019. ^*^Individual Employers had more than one option to fund their home care.

The sample had 2,052 matched observations, between PAs and IEs, where PAs answered whether they had taken time off work due to sickness. We excluded from this sample observations with no data on variables of interest including distance to work, pay, number of jobs, tenure and hours of work (leaving *n* = 1,055). We then further restricted the sample to account for outliers for sick leave (more than 25 days), basic pay rates (> £15 per hour), distance to work (> 50 miles), age (only including PAs aged 16–90) and hours of work (< 40 hours a week), providing a final sample of 1,016 observations in our regressions.

## Methods

We estimated the following model of sick leave:


(1) $$\begin{array}{l} Sick\;leave=f\left(\alpha ,\gamma ,\delta ,\theta ,\upsilon \right) \end{array}$$

where taking sick leave, measured as either a binary yes/no measure of having at least one spell of sick leave in the last 12 months or as the total number of days of sick leave, is a function of the PAs' characteristics (γ), such as their personal or job characteristics; the IEs' characteristics (δ), for example, the number of PAs that they hire or the type of support they need; the Local Authority characteristics (θ), which includes the Job Seeker's Allowance rate and the supply of social care; a constant (α) and a residual error term (υ). We also included regional dummies, accounting for unobserved systematic differences between regions and clustered standard errors at LA-level to account for any similarities encountered by PAs located in the same LA (e.g. local job market conditions, transport).

We estimated this model using two specifications to account for the nature of the measure of sick leave. First, we estimated a probit regression of sick leave. The probit model calculates the probability of an event given a set of characteristics. Therefore the model is appropriate in this scenario, where the dependent variable is binary and assumes the value of 0 if no sick days have been taken and 1 if one or more days of sick leave were taken in the last 12 months. Calculating the marginal effects after the probit model yields the coefficients of each independent variable, showing the magnitude of the relationship between each one of them with the likelihood of taking sick leave.

The second specification used was tobit regression. This model used the number of days of sick leave as the dependent variable with the same set of characteristics as regressors. The tobit specification allows for the dependent variable to be limited in either its lower (left) or upper (right) values. In our case, the number of days of sick leave taken is skewed to the left and censored at zero, therefore making the use of the tobit model appropriate.

The above models were estimated with standard errors clustered at LA-level, which assumed that the amount of sick leave taken for each PA may be linked in an unidentified way within the same LA. To explore this further, we also specifically allowed for the nesting of PAs within LAs by using multi-level specifications. This assumed that the sample of PAs was drawn from a sample of LAs and to account for this a random intercept was included to allow for variation in sick leave by LA. We ran both multi-level specifications for probability of sick leave (probit) and sick days (tobit).

## Results

Table 3 presents the estimation results from the alternative specifications of the sick leave model. The first set of results, originating from the probabilistic model, is presented in the second column left to right, followed by the results of the tobit model, also known as censored regression model, in the third column. The probit results express the change in probability of having a sick day while the tobit model show a change in the number of sick days taken, on average.

**Table 3:** Estimation results.

**Variable**	**Probit (ME)**	**Tobit (sick days)**
Basic pay rate (log)	0.156 (0.129)	3.712 (2.443)
Distance from work (miles)	0.0135*** (0.005)	0.231*** (0.088)
Distance (squared)	−0.000*** (0.000)	−0.008** (0.003)
Permanent contract (yes)	0.098* (0.059)	1.333 (1.199)
More than one job (yes)	−0.031 (0.035)	−0.980 (0.649)
Years in the role	0.016 (0.001)	0.294 (0.219)
Years in the role (squared)	−0.001 (0.001)	−0.024 (0.017)
Fixed hours (yes)	0.041 (0.051)	0.898 (0.990)
Hours worked (log)	0.033 (0.021)	1.168*** (0.374)
Female (yes)	0.053 (0.042)	0.941 (0.895)
PA disability (yes)	−0.068 (0.070)	−0.857 (1.261)
Ethnicity (non-white)	0.040 (0.053)	0.796 (1.040)
Age	0.006 (0.007)	0.088 (0.136)
Age (squared)	−0.000 (0.000)	−0.02 (0.002)
More than one type of support need (IE)	0.017 (0.033)	0.089 (0.647)
IE learning disability (yes)	−0.011 (0.048)	0.022 (0.799)
**Funding (ref: direct payment)**		
Personal Health Budget	−0.081 (0.058)	−1.861* (1.080)
Self-funded	0.016 (0.042)	−0.206 (0.782)
Total number of PAs employed	0.029*** (0.007)	0.511*** (0.160)
IE is 65 years or older	−0.037 (0.025)	−0.287 (0.489)
JSA allowance (rate of uptake)	−0.007 (0.049)	−0.692 (0.880)
Total # home care providers	−0.002*** (0.001)	−0.037*** (0.012)
Total # of Care Homes	0.001*** (0.000)	0.014** (0.005)
**Constant**		−18.498
Pseudo *R*^2^	0.075	0.032
Observations	1,016	1,016

ME, Marginal effects; IE, Individual employer; PA, Personal Assistant; JSA, Job Seekers Allowance—unemployment benefit. Local Authority clustered standard errors are in parentheses. ***p < 0.01, **p < 0.05, *p < 0.1. Model includes controls for region.

Both models show a statistically significant positive correlation between distance to work and likelihood of taking sick leave or an increase in the number of days of sick leave. An increase of one mile in the distance to work leads to a 1.3% point increase in probability of taking sick leave according to the probit model. Similarly, the tobit model predicts an increase of 0.2 days of sick leave taken, on average. This effect fades very slowly the greater the distance, as shown by the squared distance variable, meaning that each further increase in the distance yields slightly smaller effects. Based on the coefficients for distance and its quadratic value in the tobit model, the greater the distance from work the greater the number of sick days up to a distance of 15 miles.

In terms of other job characteristics, the estimation results show there was no significant effect of wage in either models. There was some evidence that having a permanent contract was positively associated with taking sick leave, however this was not captured in the tobit model. Longer hours worked also had a positive association with sick leave in the tobit model as an increase of 1% in hours worked increased the average number of days of sick leave taken by almost 1.2. However, there was not a significant relationship in the probit model.

There was no evidence of association of domestic workers' gender, ethnicity, being disabled or the number of other jobs they had and sick leave. From the Individual Employers (IE) perspective, funding the payment of PAs through NHS's PHB seemed to play a role in reducing by 1.9 the number of days of sick leave taken. The number of PAs employed by the IEs also had a noticeable impact on taking sick leave, with each extra employee increasing the chances by 2.9% in the probit model, and by 0.5 days of sick leave in the tobit model.

Taking into account Local Authority (LA) characteristics, the unemployment rate was shown to have a negative association with absenteeism, but in neither model was this association significant. There was also evidence that the number of home care providers had a significant negative association with sick leave.

The results of the multi-level models, which allowed for variation in probability of sick leave and sick days by LA, are reported in Table 4. We found that the variation between LAs in probability of taking sick leave and sick days to be above zero but extremely small (< 0.001%) and did not alter the main findings from the linear models. Additionally, in the multi-level tobit model, we found weak evidence for sick days having an association with both hourly wage and local unemployment. Finally, as a robustness check, we allowed distance to vary in size of effect across LAs. We again found very little difference in the size of effect of distance on probability of sick leave and sick days across LAs (< 0.001% variance).

**Table 4:** Multilevel estimation results.

**Variable**	**Probit (ME)**	**Tobit (sick days)**
Basic pay rate (log)	0.205 (0.128)	4.451* (2.400)
Distance from work (miles)	0.014*** (0.005)	0.243*** (0.085)
Distance (squared)	−0.000*** (0.000)	−0.008*** (0.003)
Permanent contract (yes)	0.095 (0.060)	1.286 (1.194)
More than one job (yes)	−0.037 (0.035)	−1.076* (0.639)
Years in the role	−0.017 (0.011)	0.296 (0.217)
Years in the role (squared)	−0.001* (0.001)	−0.024 (0.016)
Fixed hours (yes)	0.044 (0.050)	0.889* (0.973)
Hours worked (log)	0.033 (0.020)	1.148*** (0.365)
Female (yes)	0.054 (0.042)	0.905 (0.896)
PA disability (yes)	−0.071 (0.070)	−0.924 (1.272)
Ethnicity (non-white)	0.039 (0.047)	0.876 (0.941)
Age	0.006 (0.007)	0.093 (0.137)
Age (squared)	−0.000 (0.007)	−0.002 (0.002)
More than one type of support need (IE)	0.016 (0.033)	0.112 (0.651)
IE learning disability (yes)	−0.014 (0.048)	−0.030 (0.798)
**Funding (ref: direct payment)**		
Personal Health Budget	−0.085 (0.058)	−1.961* (1.086)
Self-funded	0.018 (0.041)	−0.233 (0.781)
Total number of PAs employed	0.031*** (0.007)	0.520*** (0.161)
IE is 65 years or older	−0.043* (0.025)	−0.383 (0.470)
JSA allowance (rate of uptake)	−0.066 (0.046)	−1.498 (0.787)
Total # home care providers	−0.002*** (0.000)	−0.025* (0.010)
Total # of care homes	0.001*** (0.000)	0.009* (0.005)
Constant		−18.476
Wald test	104.91***	93.73***
Pr>Chi^2^	0.000	0.000
Variance across LAs (_constant)	1.9e^−33^	6.6e^−33^

ME, Marginal effects; IE, Individual employer; PA, Personal Assistant; JSA, Job Seekers Allowance—unemployment benefit. Robust standard errors are in parentheses. ***p < 0.01, **p < 0.05, *p < 0.1. Both models had 1,016 observations.

## Discussion

### Quantitative findings, limitations and strengths

We used the Skills for Care survey of Individual Employers and Personal Assistants collected in 2019 in England to explore the reasons for PA sick leave. Data was available for more than 2,000 PAs and included information on their employers' funding and needs. We used linear and multi-level models to estimate both the probability of taking sick leave and the number of sick days a PA had. This article fills a gap in the literature by providing empirical evidence using econometric methods in a topic that has tended to be researched using qualitative methods.

Our results provide evidence that PAs' job, employer and local area characteristics all affected taking sick leave. We found that PAs who had a permanent contract were more likely to take sick leave. A possible explanation is that by having a permanent contract the employees have better job security and feel more able to take sick leave or potentially a permanent contract may have better working conditions attached to it such as (paid) sick leave.

There was a significant association indicating that IEs using PHBs to pay their PAs faced lower absenteeism from their employees. This evidence is weak and differs from the analysis of Gousia and Allan (25) on turnover using the same SfC survey. As discussed by the authors of that study, people receiving PHB have additional health care needs on top of social care needs, which makes it more difficult to find PAs with the proper mix of skills leading to higher turnover/vacancies. However, the results presented here suggest that even though this might be the case, once there is a good match between PAs and IEs then the absenteeism is lower as a reflection of that good match. It could also indicate greater job satisfaction from greater training opportunities/increased skills in dealing with health related tasks.

Looking into local characteristics, a negative association between unemployment rate and absenteeism only found to be weakly significant in one model. Previous literature has generally found a negative association between unemployment and turnover (25, 49–51). Exploring further local characteristics, our results did indicate that greater availability of home care providers reduced absenteeism. This result could indicate that PAs have to consider a potential substitution effect from too much absence, i.e. independent employers utilizing home care agencies with their direct payments instead. If so, this will likely be a better measure of alternative care provision for employers than local unemployment rates. Conversely, the number of local care homes had a small but positive association with sick leave, similar to results from a previous study (25). This finding suggests care homes act as a potential alternative employer for PAs.

Besides the results discussed previously in this section, our results showed a strong positive association between the distances that PAs have to travel to work, the number of PAs hired by IEs and increased absenteeism. Travel time to work can be seen as a measure of job quality and satisfaction, so longer distances might contribute to lower job satisfaction, while the greater number of PAs employed could be indicative of a caring motive, with PAs looking to avoid leaving the IE without care.

We also found weak evidence in one model of sick days that higher basic pay is positively associated with taking sick leave. This could indicate that PAs on better wages are more able to take sick leave or is a proxy for overall better pay, potentially including sick pay. However, one of the limitations in our study is that we were not able to control for unobserved heterogeneity and this might be the reason why we did not find a strong relationship between basic pay and taking sick leave as found in another study (46). Future research could look to extend this work utilizing appropriate methods to address this. Another limitation is the question regarding how representative is the sample used in our study. Despite our average profile for PAs matching with small samples from other studies discussed previously, the lack of other independent sources of large datasets on PAs hinders our assertion that the sample used in this study is representative and this could potentially affect the results found. However, we noted above that the survey had good coverage of PAs across the country and is the best source of information currently available.

### Involvement of research advisors

This study had the active involvement of two research advisors: Eleni Chambers (EC) and Debs Smith (DS). Following the project start in June 2021, EC and DS responded to an advert for advisor involvement that was sent to members of the PSSRU Public Involvement Research Advisor Network (PIRAN) group. Both EC and DS joined the study team shortly afterwards. There have been several meetings during the course of the study where, as people with lived experience of the subject, EC and DS have commented on the aims and methods of the study, provided lay interpretation to the results and been involved in dissemination activities. The study followed the National Institute for Health and Care Research (NIHR) Center for Engagement and Dissemination (CED) guidelines on pay and other practicalities (52).

During a first meeting to introduce the study, we discussed how the research advisors would best like to be further involved in the project, subject to budget constraints. Both advisors were keen to be involved in dissemination of findings. In a follow-up meeting on research advisor participation in dissemination, EC and DS had a preference for working as co-authors on a journal article of study findings. We discussed the different ways in which they could be involved in providing input as co-authors and a preferred methodology was developed where both research advisors would reflect on the findings of the study through reflective answers to questions posed on the study findings by the rest of the study team. This discussion had the primary aim of providing a local and national context to the findings given their limited knowledge of international long-term care. In addition to this discussion, EC and DS also read and provided comments on all sections of the paper.

Overall, EC and DS contributed to the study by providing a real-life context and previous research advisor experience which (1) enabled the study team to critically assess and alter the preliminary model of sick leave; (2) helped in interpreting and dissemination of findings; and (3) further developed study team members' skills and knowledge of involving those with lived experience in research.

### Research advisor reflection on study findings

- **What is your lived experience of social care?**

EC: I have used LTC services and had a Direct Payment since 2009. I initially used Council provided services, then private agencies and since 2015 have employed Personal Assistants (PAs). Even though there is more work for me to do, in terms of the recruitment and selection, training of PAs and paperwork involved in managing them, I much prefer being an individual employer because it affords me greater choice and control.

DS: I have for a number of years supported someone who has employed a personal assistant and they have paid for that PA through direct payments they receive. Before they were awarded direct payments they had had a short time of carers who were sent from a care agency that the social care providers used. This meant having no continuity and was difficult. As it so happens there was a time when the long term health conditions I have meant that I had care in this way too.

- **What was your involvement in this study?**

EC: Debs (DS) and myself have been involved in this study since June 2021. We have discussed and commented on the aims of the study, assisted with interpreting the results and been involved in dissemination. We've had an opportunity to be involved in other areas, this article for example, and I've appreciated the flexibility of the research team and their commitment to involvement.

DS: I have given a lot of support as an informal carer to my friend in recruiting and employing over time now 4 PAs and it had not been easy. It was as a result of this experience that I got I involved in this study as someone with lived experience of the subject and I have worked as an advisor in this capacity on the study having a number of meetings with the researchers and learning about the study and its results and using my lived experience to reflect and guide the study and make comments on its findings.

- **How do the findings of this study fit with your experience of the employment of personal assistants?**

EC: I always employ at least two PAs which enables one to cover for the other during periods of sickness or holidays. My Direct Payment is only relatively small so they both only work a few hours each week and because of this I tend to recruit students as I find that these hours fit well round their studies and I am able to be flexible regarding the days they work. Sometimes PAs are studying a Health and Social Care course so working as a PA provides them with valuable work experience in a related field. Usually, PAs stay for 2 or 3 years with me, often until their course ends, although some people have stayed longer.

As most of my PAs don't have cars themselves, how far away they live has always been an influential factor. Most of them have used public transport and a convenient bus route has always been helpful. Some have used the journey to and from work as an opportunity for exercise—several have cycled into work and some have walked. In my experience distance to work is certainly important as this study found, however, it is also important to take into account related issues such as convenience of transport and personal preferences of PAs. My PAs have been of different genders, ethnicities and several have had disabilities or long term health conditions. I have noticed no correlation with these and sick leave. I have always used a permanent contract and pay above the hourly rate that my local Council recommends. Most of my PAs have worked no more than 8 hours/week and have been with me no longer than 3 years. Some have had other jobs, including PA roles with other people. I have observed no association between any of these factors and sick leave.

DS: The findings of the study do mirror quite a bit what has happened with the PAs my friend has had. The main difficulty has been recruiting people who lived locally and who could do the hours that for which my friend had funding. Another difficulty was my friend not coping with all the paper work that needed to be done and having to get permission for me to do it for her. I feel that now we have these results from a robust research study we need to use them to make changes to the way PAs are viewed, trained and employed.

- **How can these findings help with the recruitment and retention of personal assistants?**

EC: What seems most important to me when employing PAs is providing good working conditions—this includes developing and maintaining a positive culture of trust, respect, integrity, equality and flexibility. Where possible, I also try to tailor the work that each PA does to fit in with their skills and interests.

DS: If there were more funding per hour for the PAs, if they had more time with people for the work they had to do and there was a way of them being trained that could equip them in time to progress to other jobs then this would reduce the stress on PAs generally and help them feel more valued. This would also help them feel more valued and could well lead to less sickness and absenteeism.

- **What more can be done to help the recruitment and retention of personal assistants?**

EC*:* As always, local and national government can enable greater flexibility in how personal budgets, including the employment of PAs, can be spent—I find myself restricted at times in what I am allowed to spend the budget on. Not only that, PAs could be carrying out more interesting and varied tasks but are not able to do that at present due to restrictions.

The lack of support for IEs is often an issue. My local Disabled People's User Led Organization (DPULO) facilitates some excellent training and support initiatives, and they also enable me to meet other people with personal budgets—arch to explore these factors in greater depth. I would particularly like to see research led by disabled people themselves or co-produced with academics, and also greater use made of gray literature.

DS: The other thing that policy makers need to look at to help with this problem is giving much more support to those who need PAs, or their loved ones, in the process of recruiting and employing the PAs generally. That way we can ensure that we get the right people into the jobs of PAs and that those unsuited to the role are not employed, as the inexperience of service users and their loved ones in doing the recruiting and employing of PAs in the first place could be contributing to the problem of sickness and absenteeism. I would recommend further more qualitative research is done where PAs and their employers can be interviewed to try and find out what number of those involved in these positions say lies behind the sickness and absenteeism of PAs and what could be done about it. I would also recommend that future studies look at what happens in a number of other countries to see what can be learnt from them.

## Conclusion

This study looked into the factors associated with work absenteeism of PAs in England. This market has flourished since legislation passed in recent decades allowed LTC users to hire assistants directly, leading to a growth in the number of PAs in England. Similarly, in other countries, the aging population and the increasing need to address LTC demands will most likely see an increase in the size of that market in the coming years and decades, highlighting the importance of understanding the reasons why PAs take sick leave from work, as it might be an indicator of future turnover and vacancies which are known to be associated with poorer outcomes for LTC users.

Using data from a survey of PAs, which included personal, job and employer information, we found evidence that job characteristics, such as travel distance to work and hours of work, and local area characteristics including social care supply, had significant associations with taking sick leave.

These findings have important policy implications for long-term care and for the recruitment and retention of domestic care workers in particular. The results indicated strong evidence of the local nature of the market for PAs, with travel distance to work and local economy factors (alternative LTC employers/providers) significantly influencing the likelihood of a PA taking sick leave. This provides policymakers with evidence that local issues can influence employment in LTC and that policy should be locally focused. For example, having an easy to access public transport service might play a role in promoting PA recruitment and retention, and this may particularly apply to rural areas. Additionally, a recruitment strategy that works in a neighborhood or town with low social care employment may not work in a neighboring location with high social care employment. Linked to the above, we also found evidence of the interlinked nature of employment in LTC. Recruitment and retention strategies could look to utilize this evidence to promote careers in LTC, e.g. encouraging students to work as PAs with a clear career path available beyond this as they graduate and begin full-time employment. Overall, the results found are an attempt to fill a gap in the literature regarding absenteeism of PAs in England and elsewhere, a stepping-stone for future studies with more complete datasets and robust estimation strategies, and also qualitative studies that can disentangle the effects found.

Our study also had active involvement of two research advisors with first-hand experience of social care use who contributed with a real-life context of PA recruitment and retention issues and previous experience as research advisors. The discussion of results in the light of this experience was confirmed with anecdotal impressions, providing explanations to the results and pathways to explore in the future. A potential limitation to this section was that we could not include the views of a PA and this would be something to add to future work in this area. Nonetheless, the participation of people with lived experience of employing PAs in this study was innovative and shows a commitment to integrate academic studies with its stakeholders in accordance with NIHR guidelines (53), facilitating guidance on the direction of research, the dissemination of knowledge and the debate regarding public policies and personal initiatives that are best tailored to the individuals who need it.

## Data availability statement

The data analyzed in this study is subject to the following licenses/restrictions: The Individual Employer and Personal Assistants survey 2019 dataset is available upon request to the Skills for Care organization. Requests to access these datasets should be directed to Skills for Care, analysis@skillsforcare.org.uk.

## Author contributions

Conceptualization of research and review of manuscript draft: DR, SA, EC, DS, and KG. Methodology development and data analysis: DR and SA. Data cleaning: DR and KG. Original manuscript draft: DR, SA, EC, and DS. All authors contributed to the article and approved the submitted version.

## Funding

This study was part of the Retention and Sustainability of Social Care Workforce (RESSCW) Project (award reference number: 1325587) funded by the Health Foundation's Efficiency Research Programme. The Health Foundation is an independent charity committed to bringing about better health and health care for people in the UK. We also thank Skills for Care for their very helpful support with using the Survey of Individual Employers and Personal Assistants.

## Conflict of interest

The authors declare that the research was conducted in the absence of any commercial or financial relationships that could be construed as a potential conflict of interest.

## Publisher's note

All claims expressed in this article are solely those of the authors and do not necessarily represent those of their affiliated organizations, or those of the publisher, the editors and the reviewers. Any product that may be evaluated in this article, or claim that may be made by its manufacturer, is not guaranteed or endorsed by the publisher.

## Author disclaimer

The views expressed in this article are entirely those of the authors.

## Footnotes

^1^Sample percentage of Local Authorities present in each region of England: East Midlands (89%), East of England (100%), London (91%), North East (83%), North West (91%), South East (74%), South West (93%), West Midlands (100%) and Yorkshire & Humber (93%).
