# Supplementary material for: Personal assistants in England and the factors associated with absenteeism
Source: Front Public Health. 2022 Oct 10;10:970370. doi: 10.3389/fpubh.2022.970370 (PMC9589044; doi:10.3389/fpubh.2022.970370)
Supplement: Supplementary file 1 [file Table_1.DOCX]

Table 5 – Robustness check with varying size of effect for distance across LAs

| **Variable** | **Probit (ME)** | **Tobit (sick days)** |
| --- | --- | --- |
| Basic pay rate (log) | 0.620 (0.388) | 4.451* (2.400) |
| Distance from work (miles) | 0.044*** (0.014) | 0.243*** (0.085) |
| Distance (squared) | -0.001*** (0.000) | -0.008*** (0.003) |
| Permanent contract (yes) | 0.287 (0.182) | 1.286 (1.194) |
| More than one job (yes) | -0.111 (0.106) | -1.076* (0.639) |
| Years in the role | 0.051 (0.033) | 0.296 (0.217) |
| Years in the role (squared) | -0.004* (0.003) | -0.024 (0.016) |
| Fixed hours (yes) | 0.133 (0.151) | 0.889* (0.973) |
| Hours worked (log) | 0.099 (0.062) | 1.148*** (0.365) |
| Female (yes) | 0.163 (0.128) | 0.905 (0.896) |
| PA disability (yes) | -0.216 (0.211) | -0.924 (1.272) |
| Ethnicity (non-white) | 0.119 (0.141) | 0.876 (0.941) |
| Age | 0.019 (0.021) | 0.093 (0.137) |
| Age (squared) | -0.000 (0.000) | -0.002 (0.002) |
| More than one type of support need (IE) | 0.047 (0.101) | 0.112 (0.651) |
| IE learning disability (yes) | -0.041 (0.146) | -0.030 (0.798) |
| *Funding (ref: Direct Payment)* |  |  |
| Personal Health Budget | -0.256 (0.175) | -1.961* (1.086) |
| Self-funded | 0.053 (0.125) | -0.233 (0.781) |
| Total number of PAs employed | 0.093*** (0.023) | 0.520*** (0.161) |
| IE is 65 years or older | -0.133 (0.076) | -0.383 (0.470) |
| JSA allowance (rate of uptake) | -0.201 (0.142) | -1.498 (0.787) |
| Total # home care providers | -0.005*** (0.001) | -0.025* (0.010) |
| Total # of Care Homes | 0.002*** (0.000) | 0.009* (0.005) |
| Constant | -2.579 | -18.476 |
| Wald test | 104.9*** | 93.73*** |
| Variance across LAs (_constant) | 3.55e^-35^ | 1.42e^-32^ |
| Variance across LAs (distance) | 1.50^e-41^ | 1.1e^-35^ |

Notes – ME: Marginal effects; IE: Individual employer; PA: Personal Assistant; JSA: Job Seekers Allowance - unemployment benefit. Robust standard errors are in parentheses. *** p<0.01, ** p<0.05, * p<0.1. Both models had 1,016 observations.
